# Alcohol Use in Adolescence: Transition from Use to Abuse

**DOI:** 10.1192/j.eurpsy.2022.160

**Published:** 2022-09-01

**Authors:** G. Dom

**Affiliations:** University of Antwerp, Psychiatry, Boechout, Belgium

**Keywords:** Transition, alcohol, abuse, adolescent

## Abstract

Understanding the factors that play a role in the initiation of alcohol use and the subsequent transition to later alcohol abuse adolescence is of paramount importance from the context of developing better-targeted types of secondary (“pro-active”) prevention interventions (Hendriks VM, Dom G., 2021). Peer and family influences together with temperament traits have been suggested to be of cardinal importance regarding the initiation of alcohol use. In addition to these factors neurobiological and genetic factors play a major role in the risk of developing alcohol abuse upon initiation. The presentation will highlight the different psychological, neurobiological, and social factors underlying the risk of the transition to abuse and dependence in adolescence. In addition, examples of targeted prevention interventions will be highlighted.

**Disclosure:**

No significant relationships.

